# Serum adiponectin and peroxisome proliferator-activated receptors-γ levels in obese patients with and without prediabetes

**DOI:** 10.1590/1806-9282.20231000

**Published:** 2024-04-22

**Authors:** Mehmet Ali Gul, Duygu Tozcu, Akın Tekcan, Mustafa Capraz, Hatice Dortok Demir

**Affiliations:** 1 Amasya University, Faculty of Medicine, Department of Medical Biochemistry – Amasya, Turkey.; 2 Amasya University, Faculty of Medicine, Department of Physiology – Amasya, Turkey.; 3 Amasya University, Faculty of Medicine, Department of Medical Biology – Amasya, Turkey.; 4 Amasya University, Faculty of Medicine, Department of Internal Diseases – Amasya, Turkey.

**Keywords:** Prediabetes, Obesity, Adiponectin, PPAR-gamma

## Abstract

**OBJECTIVE:**

Obesity is an increasingly prevalent global health problem, which is generally caused by the increase in body fat mass above normal and observed in all societies. If the blood glucose level is higher than normal but not high enough to diagnose diabetes, this condition is defined as prediabetes. Adiponectin increases fatty acid oxidation and insulin sensitivity and is closely associated with obesity. One of the nuclear receptor superfamily member peroxisome proliferator-activated receptors is shown to have an important role in various metabolic reactions. This study aimed to investigate the serum levels of adiponectin and peroxisome proliferator-activated receptors-gamma parameters, which are closely related to adipose tissue, energy metabolism, and insulin sensitivity, in obese patients with and without prediabetes.

**METHODS:**

For this purpose, 52 obese patients with prediabetes, 48 obese patients with non-prediabetes, and 76 healthy individuals were included in this study. Serum adiponectin and peroxisome proliferator-activated receptors-γ levels were analyzed by ELISA.

**RESULTS:**

Serum adiponectin levels were significantly higher in obese patients with prediabetes (18.15±15.99) compared with the control group (15.17±15.67; p=0.42). No significant difference was observed in both adiponectin and peroxisome proliferator-activated receptors-γ levels in the obese patients with the non-prediabetes group compared with the control group. However, no significant difference was observed in the obese patients with prediabetes group and obese patients with non-prediabetes group.

**CONCLUSION:**

Our results suggest that adiponectin may serve as an indicator of prediabetes. This implies that examining adiponectin levels in individuals diagnosed with prediabetes may enhance our understanding of the metabolic processes closely linked to prediabetes and related conditions.

## INTRODUCTION

Obesity is a disease characterized by an increase in the ratio of body fat to the whole body, defined by a body mass index of 30 kg/m^2^ or greater (weight divided by the square of height). The prevalence of obesity is increasing day by day and its prevalence has reached a pandemic level worldwide^1^.

Although it is inevitable for patients with prediabetes to be type 2 diabetes mellitus (T2DM), some prediabetes do not develop diabetes and it is estimated that the annual conversion rate of prediabetes to diabetes is 5–10%^2,3^. Therefore, it is important to investigate whether prediabetes, which is one of the T2DM developmental stages of obesity, is a causal risk factor^4^.

Metabolic disorders that start with insulin resistance in obesity can cause prediabetes. Prediabetes clearly increases the risk of T2DM. Although it is an important health problem, most prediabetics are not aware of their prediabetes^5^. Adipokines are secreted from adipose tissue and play a role in many physiological events, such as lipid metabolism, glucose metabolism, and inflammation, which are closely related to obesity and prediabetes^6,7^. Peroxisome proliferator-activated receptor gamma (PPAR-γ) is a regulatory nuclear protein found mainly in adipose tissue, has anti-inflammatory properties, increases insulin sensitivity, and has important effects on adipocyte proliferation and cell cycle control^8,9^. Adiponectin and PPAR-γ associated with obesity and diabetes have a direct effect on lipid metabolism, insulin sensitivity, and glucose-energy metabolism. In light of this information, this study aimed to investigate the serum levels of adiponectin, which is an important member of the adipokine family, and PPAR-γ, which is an important member of the nuclear receptor family, on prediabetes and obesity.

## METHODS

A total of 52 obese patients with prediabetes, 48 obese patients with non-prediabetes, and 76 healthy individuals who applied to Amasya University Sabuncuoğlu Şerefeddin Training and Research Hospital Internal Diseases Department were included in the study. Anthropometric measurements of the individuals were included in the study during their application to the outpatient clinic. At the same time, the patients' age, gender, different drug use, diet, smoking, alcohol, and other disease anamnesis were taken. The blood samples taken from the patients who applied after at least 8–12 h of fasting were routinely examined. Blood samples were taken for routinely requested serum samples, and 5 mL of blood was collected in yellow-capped gel tubes. It was slowly turned upside down 5–6 times. After waiting for at least 30 min, it was centrifuged at 1500–2000×g for 10 min with a centrifuge device. The serum part, which was separated at the top of the tube, was aliquoted and transferred to the Eppendorf tubes. The transferred samples were stored in a deep freezer at −80°C until the working day. Adiponectin and PPAR-γ levels were determined from the stored serum samples by using the ELISA method. ELISA analyses were performed according to the kit procedure instructions. The study was approved by the Ethics Committee of Amasya University (03/2021 Decision Number:6/100) and performed in accordance with the ethical standards specified in the Declaration of Helsinki.

Serum adiponectin and PPAR-γ levels were determined using the Human Adiponectin and Human PPAR-γ ELISA kit (Bioassay Technology Laboratory, Birmingham, UK) according to the manufacturer's instructions. Serum adiponectin and PPAR-γ measurements were performed using the Chromate 4300 Elisa reader (Awareness Technology, Inc., Martin Hwy., Palm City, USA).

Statistical analysis of the data was carried out using Statistical Package for the Social Sciences version 20.0 (IBM SPSS Corp., Armonk, NY, USA). Whether the data were normally distributed was evaluated using the Kolmogorov-Smirnov and Shapiro-Wilk tests. Also, the skewness and kurtosis values were analyzed. The Mann-Whitney U test, which is one of the nonparametric tests, was used for the data that did not comply with the normal distribution. The results were given as mean±standard deviation (mean±SD), p-value below 0.001 were contemplated very significant, and p-value below 0.05 were contemplated statistically significant.

## RESULTS

There was no statistical difference between the control, obese patients with non-prediabetes, and obese patients with prediabetes group in terms of age (years) (50.13±9.49 vs. 46.34±10.50 vs. 50.88±9.58), height (cm) (160.29±11.68 vs. 161.25±10.73 vs. 159.72±10.32), smoking, alcohol use, and other diseases. A statistical difference was determined between control, obese patients with non-prediabetes, and obese patients with prediabetes group considering the presence of weight (kg) (80.53±14.81 vs. 94.52±17.70 vs. 86.65±12.89; p<0.001), BMI (31.69±6.92 vs. 36.27±5.40 vs. 34.11±5.41; p<0.001), and waist circumference (cm) (98.14±10.91 vs. 106.19±11.14 vs. 103.43±8.24; p=0.001), which were found to be statistically high in the obese patients with non-prediabetes group (Table 1).

**Table 1:** Baseline clinical and demographic features of the patients and controls.

Characteristics	Controls (n=76)	Obese patients with non-prediabetes (n=48)	Obese patients with prediabetes (n=52)	p-value
Gender, F/M, n (%)	56/20 (73.7/26.3)	36/12 (75.0/25.0)	33/19 (63.5/36.5)	
Age, mean±SD, years	50.13±9.49	46.34±10.50	50.88±9.58	
Adiponectin (ng/mL), mean±SD	15.17±15.67	16.63±15.64	18.15±15.99	**0.023** *
PPAR-γ (ng/mL), mean±SD	3108.33±2932.72	3244.16±3324.42	2950.45±2490.87	0.885
PPAR-γ/adiponectin ratio	259.32±208.38	206.44±114.71	206.57±229.66	**0.047** **
Height (cm), mean±SD	160.29±11.68	161.25±10.73	159.72±10.32	0.786
Weight (kg), mean±SD	80.53±14.81	94.52±17.70	86.65±12.89	**0.000** **
BMI, mean±SD	31.69±6.92	36.27±5.40	34.11±5.41	**0.000** **
Waist circumference, mean±SD	98.14±10.91	106.19±11.14	103.43±8.24	**0.001** **
Smoking, yes/no, n (%)	12/64 (15.8/84.2)	7/41 (14.6/85.4)	6/46 (11.5/88.5)	
Alcohol, yes/no, n (%)	0/76 (0.0/100.0)	0/48 (0.0/100.0)	2/50 (3.8/96.2)	
Other diseases, yes/no, n (%)	21/39 (35.0/65.0)	27/14 (65.9/34.1)	25/22 (53.2/46.8)	

F: female; M: male; SD: standard deviation; DM: diabetes mellitus; BMI: body mass index.

* Between the control group and obese patients with prediabetes group.

** Between all groups. Statistically significant p-value are denoted in bold.

There was no statistical difference between the obese patients with prediabetes (2950.45±2490.87) and obese patients with non-prediabetes (3244.16±3324.42). Also, there was no statistical difference between the obese patients with non-prediabetes and control groups (3108.33±2932.72) in terms of PPAR-γ levels (ng/mL). However, there was no significant difference between serum adiponectin levels in the obese patients with non-prediabetes and control groups. Our remarkable finding was that serum adiponectin levels (ng/mL) of the obese patients with prediabetes group (18.15±15.99) were higher than the control group (15.17±15.67; p=0.42).

In addition, PPAR-γ to adiponectin ratio was statistically higher in the control group (259.32±208.38), obese patients with prediabetes group (206.57±229.66), and obese patients without prediabetes group (206.44±114.71).

**Figure 1:**
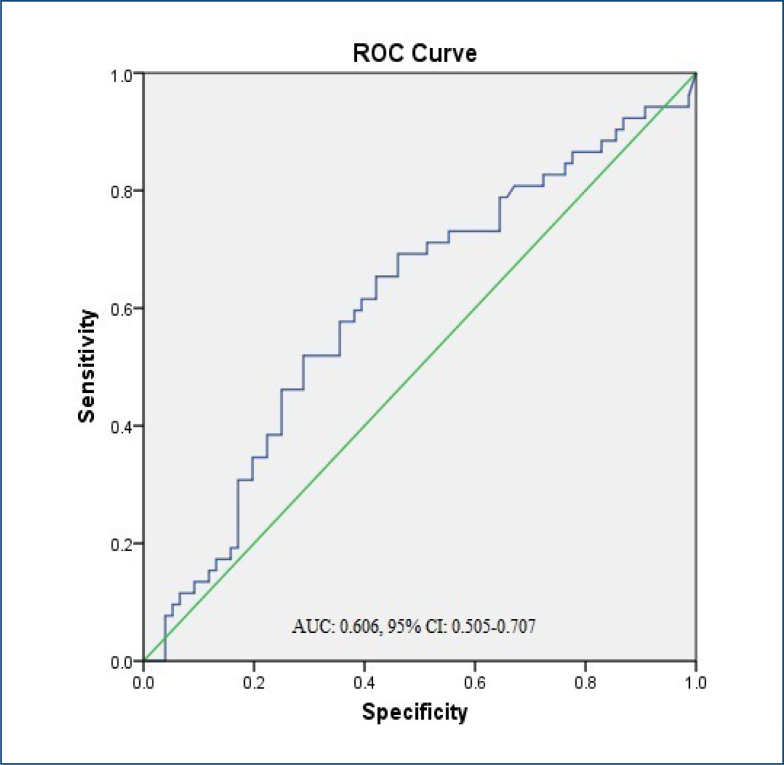
The receiver operating characteristic analysis results of adiponectin in obese patients with prediabetes compared to controls.

Receiver operating characteristic (ROC) analysis was applied for adiponectin in obese patients with prediabetes compared with controls. According to our results, the analysis of adiponectin shows low diagnostic accuracy in obese patients with prediabetes compared with healthy controls (Figure 1).

## DISCUSSION

In this study, we have shown that circulation of serum adiponectin and PPAR-γ concentrations differed based on the degree of prediabetes in obese and non-obese patients. We demonstrated that lower serum concentrations of adiponectin were present in control groups than in obese patients with prediabetes. Also, when obese patients with non-prediabetes compared with healthy non-obese patients, there were no significant differences between serum adiponectin and PPAR-γ levels. When obese patients without prediabetes were compared with healthy non-obese patients, no difference was found between serum PPAR-γ levels.

Adiponectin is a member of the adipokine family, which is secreted from adipose tissue, which is considered an endocrine organ, has antidiabetic and anti-inflammatory properties, and has functions such as insulin sensitivity, atherosclerosis, cell proliferation, and regulation of energy metabolism^10–12^. Obesity is an important public health problem and increases the risk of serious diseases such as T2DM and cancer. It is known that altered expression of adipokines affects fat accumulation in obesity. It is important to investigate this issue as adiponectin can act as a protective and safe factor to prevent obesity progression^13^.

Gateva et al., showed lower adiponectin levels in patients with prediabetes compared with those without prediabetes, and Stojanović et al., indicated that decreasing the level of adiponectin was strongly implicated in the development of insulin resistance and may be a useful marker for coronary artery disease, metabolic syndrome, and prediabetes^14,15^.

In this study, levels of serum adiponectin in obese patients with prediabetes were found statistically significantly higher than controls (p<0.42). However, there was no difference between obese patients with prediabetes and obese patients with non-prediabetes (p>0.05). Also, there was no difference between obese patients with non-prediabetes and control groups (p>0.05).

To the best of our knowledge, there are not many studies on serum PPAR-γ and adiponectin levels in obese and non-obese prediabetes. Contrary to our results, although this study is not specific to obese patients, in their meta-analysis study, Lai et al., showed that prediabetes patients had lower adiponectin levels than healthy controls, based on the lower circulating adiponectin levels before the onset of diabetes^16^. In their study among healthy adults whose parents had a history of T2DM, Jiang et al., showed adiponectin level as a strong risk marker for prediabetes and explained this by the fact that adiponectin is evident during the transition from normoglycemia to prediabetes at a much earlier stage of pathogenesis, due to its well-known association with diabetes risk^17^.

It has been shown that prediabetic people have low serum adiponectin levels^16^. The low adiponectin levels observed in obese and T2DM individuals can be explained by the fact that adiponectin increases insulin sensitivity in target tissues^18,19^.

In this study, no statistically significant difference was found in serum PPAR-γ levels between all groups (between obese patients with prediabetes, obese patients with non-prediabetes, and control groups).

In their study on obese and non-obese patients with newly diagnosed T2DM, Liu et al., showed that there was a significantly reduced serum adiponectin level in the obese T2DM group compared with the T2DM group with normal BMI^20^.

Studies on obesity have shown that PPAR-γ is an important regulator of fat cell formation and their normal functions^21^. The relationship between adiponectin and PPAR-γ is also present at the gene level, and adiponectin gene expression can be stimulated by PPAR-γ agonists^22^. Jones et al., showed that by ablating PPAR-γ from adipose tissue using a tissue-specific gene ablation approach, adiponectin gene expression was significantly reduced in adipocytes and also resulted in decreased circulating levels^23^. Treatment using a PPAR-γ agonist has been shown to increase adiponectin secretion and improve insulin resistance in rats and humans^24^. The clinical benefits of administering these agonists in preventing the progression of prediabetes to T2DM are not yet fully known. Although the irregularity of serum adiponectin levels in prediabetes may play a role in the pathogenesis of the disease, it may be better to prefer lifestyle changes to pharmacological treatment that will increase serum adiponectin levels because the results of using agonists are not known exactly^25^.

## CONCLUSION

We aimed to investigate serum adiponectin and PPAR-γ levels of both prediabetic obese and non-prediabetic obese patients in the design of our study as obesity is known to be a risk factor for the development of T2DM and prediabetes is a risk factor for the development of T2DM. As it is known, adiponectin increases insulin sensitivity in adipose tissue. Obesity and prediabetes also cause the development of T2DM as a result of metabolic disorders that begin with insulin resistance. In this context, we think that the findings of this study will contribute to the literature, as we have not encountered a study similar to the patient grouping design of our study in the literature.

Among the findings of our study, the fact that serum adiponectin levels were significantly higher in obese patients with prediabetes compared with the control group supports both our hypothesis and the literature. However, the fact that the PPAR-γ/adiponectin ratio was statistically higher in the control group compared with the prediabetic and non-prediabetic obese patient groups also supports our hypothesis. High adiponectin levels in obese patients with prediabetes may be an important marker for investigating the changes in lipid and glucose metabolism associated with both prediabetes and obesity, and even inflammatory and cardiovascular risks. If similar studies with a larger number of patients are supported, our results suggest that adiponectin may be an indicator of prediabetes, may help to understand metabolic disease processes closely related to prediabetes, and should be investigated in patients diagnosed with prediabetes.
